# Non-Instrumented Incubation of a Recombinase Polymerase Amplification Assay for the Rapid and Sensitive Detection of Proviral HIV-1 DNA

**DOI:** 10.1371/journal.pone.0108189

**Published:** 2014-09-29

**Authors:** Lorraine Lillis, Dara Lehman, Mitra C. Singhal, Jason Cantera, Jered Singleton, Paul Labarre, Anthony Toyama, Olaf Piepenburg, Mathew Parker, Robert Wood, Julie Overbaugh, David S. Boyle

**Affiliations:** 1 PATH, Seattle, Washington, United States of America; 2 Fred Hutchinson Cancer Research Center, Seattle, Washington, United States of America; 3 TwistDx Limited, Minerva Building, Babraham Research Campus, Babraham, Cambridge, United Kingdom; 4 Department of Atmospheric Sciences, University of Washington, Seattle, Washington, United States of America; Centro Nacional de Microbiología - Instituto de Salud Carlos III, Spain

## Abstract

Sensitive diagnostic tests for infectious diseases often employ nucleic acid amplification technologies (NAATs). However, most NAAT assays, including many isothermal amplification methods, require power-dependent instrumentation for incubation. For use in low resource settings (LRS), diagnostics that do not require consistent electricity supply would be ideal. Recombinase polymerase amplification (RPA) is an isothermal amplification technology that has been shown to typically work at temperatures ranging from 25–43°C, and does not require a stringent incubation temperature for optimal performance. Here we evaluate the ability to incubate an HIV-1 RPA assay, intended for use as an infant HIV diagnostic in LRS, at ambient temperatures or with a simple non-instrumented heat source. To determine the range of expected ambient temperatures in settings where an HIV-1 infant diagnostic would be of most use, a dataset of the seasonal range of daily temperatures in sub Saharan Africa was analyzed and revealed ambient temperatures as low as 10°C and rarely above 43°C. All 24 of 24 (100%) HIV-1 RPA reactions amplified when incubated for 20 minutes between 31°C and 43°C. The amplification from the HIV-1 RPA assay under investigation at temperatures was less consistent below 30°C. Thus, we developed a chemical heater to incubate HIV-1 RPA assays when ambient temperatures are between 10°C and 30°C. All 12/12 (100%) reactions amplified with chemical heat incubation from ambient temperatures of 15°C, 20°C, 25°C and 30°C. We also observed that incubation at 30 minutes improved assay performance at lower temperatures where detection was sporadic using 20 minutes incubation. We have demonstrated that incubation of the RPA HIV-1 assay via ambient temperatures or using chemical heaters yields similar results to using electrically powered devices. We propose that this RPA HIV-1 assay may not need dedicated equipment to be a highly sensitive tool to diagnose infant HIV-1 in LRS.

## Introduction

The application of nucleic acid amplification technologies (NAAT) has had a significant and beneficial impact on the diagnosis of infectious diseases. While a wide variety of molecular based technologies have been developed to accurately detect a broad range of clinically relevant pathogens in just a few hours [1], the majority of these are designed for use in fully equipped laboratories with good infrastructure, reliable electrical supply and with highly trained staff to operate them [2]–[7]. Therefore, there remains a critical need for high quality diagnostics that can be performed closer to the point of care in low resource settings (LRS) to aid in the clinical diagnosis and subsequent treatment of endemic diseases such as HIV, tuberculosis and malaria. PCR-based assays are typically used for early infant diagnosis (EID) of HIV, as they are more sensitive than other existing methods such as p24 antigen detection or reverse transcriptase activity [2]. However, the infrastructure and equipment requirements to perform PCR-based HIV diagnostics results in the need to ship specimens from rural clinics to centralized facilities for testing. In low income countries, shipping and the logistics of reporting results can delay test results for months, often contributing to loss to follow up of confirmed HIV infection [3]. The outcome for HIV infected infants who are left untreated is very poor and over 50% of HIV infected infants die within 2 years if left untreated [4]. By contrast, rapid treatment can potentially lead to a reduced viral reservoir and better outcomes, further emphasizing the need for rapid point of care diagnostics [5].

In the last decade, an expanding variety of novel NAATs have been developed to specifically meet the challenges of performing diagnostics outside of well-equipped facilities in low resource settings (LRS) [1], [6]–[9]. A particular area of research has been the development of isothermal amplification methods for nucleic acid based diagnosis. These technologies employ a uniform temperature to amplify targeted nucleic acids [1]. When compared to current PCR assays, the use of isothermal technologies reduces the need for high precision instrumentation, consistent electrical power and complex sample preparation [10], [11]. Such amplification methods include loop mediated amplification (LAMP) [12], nucleic acid sequence based amplification (NASBA) [13], cross priming amplification (CPA) [14], helicase dependent amplification (HDA) [15] and recombinase polymerase amplification (RPA) [16]. In support of these amplification technologies, several electricity dependent instruments are now available to incubate the assays and detect amplification [6], [18]–[22]. Although some of these are battery powered, they still require some degree of sustainable electrical power for recharging, and their cost (while much less than large or mid-sized real time PCR instruments) may limit their widespread adoption for use in many peripheral settings.

Cheap but robust incubation systems, such as the non-instrumented incubation amplification (NINA) platform, employ chemically derived heat for assay incubation; greatly reducing costs and increasing the potential for use at the periphery [11]. These platforms have been demonstrated to effectively amplify LAMP and HDA assays [23]–[25]. Detection of amplification products with NINA relies upon visual inspection of the test reaction for reactivity or the use of a sealed immunochromatographic strips to capture and detect hapten labeled amplicons [26]–[28]. Simple yet effective technologies like these enhance the potential to use isothermal assays and introduce high performance NAATs to the most austere environmental settings where electrical power is often sporadic or absent.

RPA is an isothermal amplification method that utilizes a recombinase to facilitate the insertion of oligonucleotide primers into their complement in a double-stranded DNA molecule [16]. The use of opposing primers allows the exponential amplification of a defined region of DNA in a manner similar to PCR. Detection of HIV RPA amplicons relies on the use of an oligonucleotide probe with a specific abasic nucleotide analogue that is recognized and cleaved by an endonuclease only when the probe is bound to its complementary target sequence [16]. The amplified products can typically be detected after only 5–20 minutes, depending on amplicon size and the template copy number and also on the sensitivity of the detection method [16]. This time to test result is significantly more rapid than other isothermal assays which generally have incubation times in the range of 45–60 minutes [12]–[15].

RPA is also unique compared to many other isothermal amplification methods in that it has an incubation temperature range similar to ambient temperatures ranges found south of 20°N in continental Africa and has been reported to amplify DNA across a broad range of incubation temperatures ranging from 25–43°C using the standard reaction conditions and formulation [16]. Moreover, in contrast to the other isothermal NAAT technologies, RPA does not primarily rely on physio-chemical dependent hybridization of oligonucleotides for functionality, but rather operates by an enzymatically driven primer-binding process. For this reason RPA does not require the reaction temperature to be precisely controlled during the reaction to be specific and an assay will tolerate comparatively large variance in the incubation temperature without apparent loss of overall sensitivity or specificity. These features translate to reduced power requirements and less stringent temperature control, further reducing the complexity and cost of an incubator. The use of simpler heating devices has been explored in previous studies. For example, recently a group reported the sensitive detection of *Plasmodium falciparum* using an RPA assay and minimal instrumentation [29].

The use of ambient temperature or simple chemical heaters for an RPA-based diagnostic has not yet been evaluated. We previously described the development of an RPA assay for rapid diagnosis of infant HIV that showed high sensitivity and specificity to the major subtypes of HIV-1 circulating globally using electrical powered, laboratory-based instrumentation [17]. In this study we investigated the potential for ambient temperatures to incubate this HIV-1 RPA assay in the absence of a reliable power source. We also analyzed meteorological data to understand the seasonal temperature fluctuations across sub Saharan Africa, the primary target market of our intended HIV diagnostic, to better understand if ambient temperatures could reliably support RPA assay use across this region. In addition, to incubate RPA assays when ambient temperatures are below 30°C, we developed a simple, low cost ($0.10 US) and reusable prototype chemical heater. Our findings in this study suggest that it is possible to develop affordable and effective incubation platforms for an RPA-based diagnostic for use in the most resource limited of settings.

## Materials and Methods

### RPA amplification and Detection

TwistAmp nfo RPA reactions were supplied by TwistDx Ltd., Cambridge, United Kingdom. HIV *pol* RPA primers and probe, described previously [17], were added to the RPA nfo assays. 10 copies of HIV DNA were added to each of 3 replicate reactions at each temperature tested. HIV proviral target DNA was from the ACH-2 cell line that contains a single full-length integrated copy of HIV-1 *Bru* (clade M, subtype B; GenBank accession number K02013.1) DNA per cell [30] and was prepared and quantified as previously described [17]. Negative template controls (NTC) contained nuclease free water in place of DNA. All TwistAmp reaction mixtures were prepared in a final volume of 50 µL as previously described [17]. Incubation was performed at the test temperature for either 20 or 30 minutes, with brief mixing by inverting 3 times at 5 minutes. The RPA nfo reactions were immediately placed on ice to slow the reaction and limit the effect of the slight differences in incubation times before the sequential addition of 5 µL EDTA (250 mM) to halt each reaction. Detection of hapten-labeled RPA amplicons was assessed with an immunochromatographic strip (ICS) fully enclosed in a cassette (BESt Cassette II; BioHelix Corp., Beverly, MA) [31]. Amplification products were also analyzed by gel electrophoresis.

### RPA incubation

To mimic various ambient temperatures, all test reaction components, RPA reagents, aluminum blocks machined to hold 0.2 mL PCR tubes, and sodium acetate trihydrate (SAT) heaters were pre-incubated for 2 hours prior to reaction set up in an environmental control chamber (Coy Laboratory Products Inc., MI, USA; or Associated Environmental Systems, Ayer, MA, USA) at temperatures between 10–44°C. Three thermocouples in each environmental chamber confirmed the temperature prior to reaction set up. Humidity in all experiments was at levels between 60–65%. The controlled incubation of RPA assays was performed using a conventional PCR thermocycler (Applied Biosystems, Foster City, CA, USA).

### Exothermal heaters

**Figure 1:**
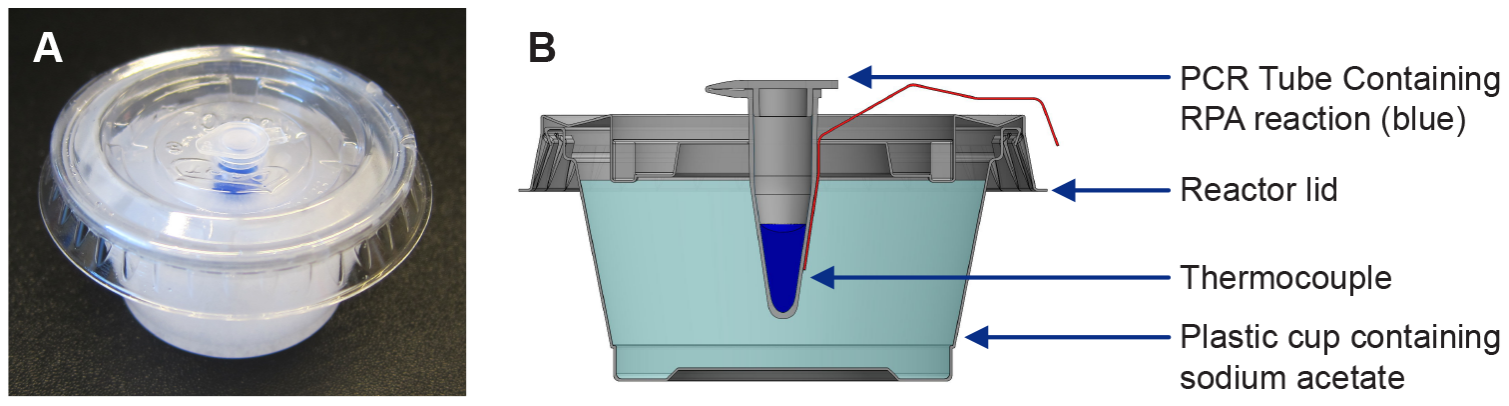
The prototype SAT heater used to incubate RPA reactions. (A) A photograph of an activated SAT heater, the reaction tube is filled with blue dye to improve definition. (B). A cross sectional diagram depicting the SAT heater and other test components.

Sodium acetate trihydrate (SAT) (Sigma Aldrich, St Louis, MO, USA) mixtures at concentrations of 70%, 72.5% and 75% were prepared and liquefied by heating to 70°C, and aliquoted into either 15 mL screw cap tubes (Fischer Scientific, Pittsburg, PA, USA) or 22 mL lidded plastic soufflé cups (Dart Container Corporation, MI, USA) with insulating foam (McMaster-Carr, Elmhurst, IL, USA) cut to fit each receptacle (Figure 1). A 5 mm diameter punch was made in the center of each cup lid to allow the insertion of a 0.2 mL PCR tube containing RPA reactions. Heat release via crystallization of the liquid SAT mixtures was induced by seeding with powdered SAT. Solidified SAT reactors were heated to 80°C for 20 minutes to reliquefy the SAT mixture, by either placing into a water bath or incubator heated to 80°C.

### Temperature measurement

Ambient temperatures and thermal profiles within the SAT solutions were measured using T-type – Physitemp IT-23 thermocouples (Omega Engineering, Everett, WA, USA) and a NI 9211 data logger (National Instruments, Redmond, WA, USA). Prior to use, each thermocouple was calibrated in boiling distilled water to verify a 100°C temperature reading on the data logger. For ambient temperature assessment within the environmental chambers, one thermocouple was positioned beside the 0.2 mL reaction tube within the tube rack, and one next to the tube rack. As a proxy for reaction temperature, an additional thermocouple was inserted through a 1.27 mm diameter hole in the lid of a 0.2 mL PCR tube filled with 50 µL deionized water which represents the RPA reaction volume. When incubating RPA reactions in the SAT heaters, a thermocouple was positioned beside the 0.2 mL reaction tube (Figure 1). The temperature was recorded at a rate of 1 data point per second for the duration of each experiment. Temperature data was further analyzed and displayed using Microsoft Excel (Microsoft Corp., Redmond, WA, USA).

### Air temperature data

A previously published bias-corrected meteorological dataset for Africa was analyzed to determine the range of daily ambient temperatures expected [32]. The dataset is derived from meteorological analyses of the physical state of the atmosphere that are produced every three hours using a weather forecasting model. The horizontal resolution of the dataset is 1×1 degree latitude/longitude (approximately 100×100 km) [33]. Air temperature data are bias and elevation corrected with quality-controlled meteorological data from stations in Africa, and the diurnal cycle of surface air temperature is adjusted to provide the best match with meteorological station data [32]. These corrections remove a bias of 2–4°C in surface air temperature over the African continent. The dataset is available for the years 1948–2010, but we use data only from the period 2000–2010 because the statistics were found to be insensitive to the inclusion of additional years.

## Results

### HIV-1 RPA assay performance at temperatures from 15°C to 44°C

To determine the range of temperatures that our HIV-1 RPA assay can tolerate, we tested the ability of this specific RPA assay to amplify 10 copies of HIV DNA at a range of temperatures from 15°C to 44°C using ICS cassettes to detect RPA amplicons. Initially, we assessed this temperature range under precisely controlled conditions using a thermocycling machine. For assays incubated for 20 minutes at ≤30°C, performance of the HIV-1 RPA assay on 10 copies of HIV-1 proviral DNA was not optimal, with only 3 temperatures producing any positive results (1/3 at 15°C and 29°C, or 2/3 at 30°C), and all 3 replicates failing at temperatures between 20°C and 27°C (Table 1). The lower incubation temperature reduces enzyme activity leading to limited amplicon formation after 20 minutes and is not be enough to be detected by LFS; however as shown by the 1 positive sample in the reactions incubated at 15°C there is remains an occasional possibility of sufficient product being amplified for detection. When incubation times were increased to 30 minutes, performance improved at temperatures between 27°C and 30°C, with all 3 replicates being positive for each temperature, but not at temperatures of 25°C and below (data not shown). Even longer incubation times than 30 minutes, which may have resulted in positive RPA signals at low temperatures, have not been tested in this study. Incubation temperatures between 31°C to 43°C provided optimal conditions in which all 3 replicates were positive at each of the 8 temperatures tested (24/24, 100%; Table 1). When increased to 44°C, performance was impaired with only 1 of 3 reactions positive. There were no false positives at any of the temperatures tested (0/15, Table 1). Gel electrophoresis was also performed on reactions from each temperature (Data not shown). Amplification products of the expected size (∼150 bp) were observed in all positive reactions heated at temperatures from 27°C–44°C. No amplification products were observed in reactions that gave negative results incubated below 27°C. This indicates that the negative results observed at lower temperatures were due to limited or no production of RPA amplicons presumably due to reduced enzyme activity and not due to competition from non-specific product formation.

**Table 1:** A comparison of the performance of HIV RPA assays with reaction incubation in either a thermocycler or via ambient air temperature.

Temp (°C)	Thermocycler				Ambient Temperature			
	HIV		NTC		HIV		NTC	
	20′	30′	20′	30′	20′	30′	20′	30′
15	1/3	0/3	0/1	0/1	0/3	0/3	0/1	0/1
20	0/3	0/3	0/1	0/1	0/3	0/3	0/1	0/1
25	0/3	0/3	0/1	0/1	0/3	0/3	0/1	0/1
27	0/3	3/3	0/1	0/1	0/3	0/3	0/1	0/1
29	1/3	3/3	0/1	0/1	0/3	2/3	0/1	0/1
30	2/3	3/3	0/1	0/1	0/3	3/3	0/1	0/1
31	3/3	3/3	0/1	0/1	3/3	3/3	0/1	0/1
33	3/3	3/3	0/1	0/1	3/3	3/3	0/1	0/1
35	3/3	3/3	0/1	0/1	3/3	3/3	0/1	0/1
37	3/3	3/3	0/1	0/1	3/3	3/3	0/1	0/1
39	3/3	3/3	0/1	0/1	3/3	3/3	0/1	0/1
40	3/3	3/3	0/1	0/1	3/3	3/3	0/1	0/1
42	3/3	3/3	0/1	0/1	3/3	3/3	0/1	0/1
43	3/3	3/3	0/1	0/1	3/3	3/3	0/1	0/1
44	1/3	0/1	0/1	0/1	2/3	2/3	0/1	0/1

20′–20 minutes total incubation time; 30′–30 minutes total incubation time; NTC - no template control.

We next tested our HIV RPA assay using an environmental chamber to simulate reactions incubated at ambient temperatures between 15°C and 44°C. Results were very similar to the thermocycler incubation results. Incubation for 20 minutes at temperatures ≤30°C were suboptimal, with only 2 of 3 reactions at 29°C, and 0 of 3 reactions positive at all other temperatures ≤30°C (Table 1). When incubation times were increased to 30 minutes, 3 of 3 reactions performed at 30°C were positive, with all other results unchanged (data not shown). Incubation at ambient temperatures between 31°C and 43°C produced consistent results with all replicates positive in this temperature range (24/24, 100%). At 44°C, performance was once again suboptimal, with only 2 of 3 reactions positive (Table 1).

### A non-instrumented heat source for incubation of RPA when ambient temperatures are ≤30°C

The previous data suggests that an external heat source is necessary for the HIV-1 RPA assay used in this study when the ambient temperature is below 30°C. We investigated the potential to use a rechargeable, exothermic material commonly used in hand warmers, a mixture of sodium acetate trihydrate (SAT) and water. A variety of SAT concentrations (from 50% to 80% w/v in water) were assessed for the temperature profiles produced after their activation from an ambient temperature of 22°C (Table S1). This preliminary screen established that SAT concentrations of 70%–75% produced heat profiles that were above 31°C but did not exceed 45°C.

**Figure 2:**
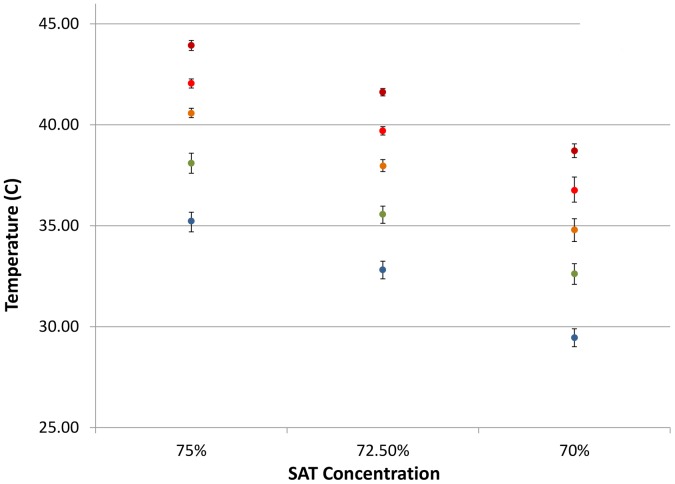
Median peak temperatures produced by different concentrations of SAT when held and activated under a variety of ambient temperatures. Ambient temperatures of SAT mixtures during activation: blue 10°C; green 15°C; orange 20°C; turquoise 25°C and red 30°C; SAT, Sodium acetate trihydrate.

Next, simulated assay temperature was measured for 30 minutes after activation of 8 mL volumes of 70%, 72.5% and 75% SAT solutions that had been stored for ≥2 hours prior to activation at ambient temperatures of 10°C, 15°C, 20°C, 25°C and 30°C (Figure 1). Each SAT solution was activated 32 times in individual tests at all of the ambient temperatures under consideration with average peak temperatures produced as shown in Figure 2. Maximal temperature was generally reached within 1 minute of the crystallization of the SAT mixture. The range of temperatures generated by each mixture did not exceed 45°C even when the heaters were conditioned to a simulated ambient temperature of 30°C prior to activation. However, when the ambient was reduced to 10°C, the 70% mixture failed to reach 30°C and was therefore not tested for incubation of RPA reactions (Figure 2).

To test the ability to use SAT heaters to incubate the HIV-1 RPA reaction, we identified small plastic tubs that allow the RPA reaction mixtures to be placed close to the center of the activated SAT mixture (Figure 1). We assessed the performance of 72.5% and 75% SAT heaters, with and without insulation, to incubate HIV RPA assays from ambient temperatures of 10°C, 15°C, 20°C, 25°C and 30°C. Nine of 10 of the insulated reactions reached 95% of the maximum recorded incubation temperature in under 15 seconds (range 4 to 18 seconds, Table 2). The non-insulated reactions took longer to reach the maximum temperatures recorded, with a range of 5 to 85 seconds. At ambient temperatures of 15°C and above, insulated 75% SAT heaters rapidly generated sufficient heat to provide incubation for 30 minutes maintaining the 31–43°C temperature range required for the HIV-1 RPA assay (Table 2). At the lower ambient temperature of 10°C, the temperature fell to below 30°C in less than 10 minutes (Table 2). The 72.5% SAT heater was comparable above at ambient temperatures above 15°C, but again also did not maintain the minimal required temperature of 31°C after 20 minutes at ambient temperatures ≤15°C.

**Table 2:** Thermal profiles of the heat generated via the insulated and non-insulated SAT heaters under a range of ambient storage temperatures.

	72.5% SAT										75% SAT									
Ambient storage temp. (°C)	10		15		20		25		30		10		15		20		25		30	
Insulation	Yes	No	Yes	No	Yes	No	Yes	No	Yes	No	Yes	No	Yes	No	Yes	No	Yes	No	Yes	No
Maximum Temp. (°C)	33	35	37	39	37	40	40	41	41	43	33	34	37	34	40	38	43	37	43	41
Temp. (°C) at 10 minutes	31	26	34	30	36	33	39	38	40	42	31	25	35	26	39	32	41	35	43	40
Temp. (°C) at 20 minutes	29	17	31	22	34	27	38	34	39	40	27	17	32	20	37	27	40	32	42	38
Temp. (°C) at 30 minutes	27	13	28	21	32	24	37	31	38	37	25	13	30	18	35	24	39	30	41	36
Temp. loss over 30 minutes (°C)	6	22	9	18	5	16	3	10	3	6	8	21	7	16	5	14	4	7	2	5
Time (sec) to reach max. temp.	10	82	123	89	94	57	123	111	14	287	80	52	109	53	111	71	20	240	182	238
Time (sec) to 95% of max. temp.	4	8	18	11	5	15	5	5	4	85	6	7	12	7	5	10	4	39	5	28

SAT, Sodium acetate trihydrate.

Insulated SAT reactors containing 72.5% and 75% SAT were then assessed for their ability to incubate the HIV RPA assays at a range of ambient temperatures from 10°C–30°C in 5°C increments. As before, triplicate HIV-1 RPA assays were performed on 10 copies of HIV-1 DNA at each starting ambient temperature and SAT concentration. All 12 of 12 (100%) reactions at ambient temperatures between 15°C and 30°C were positive when incubated by either activated SAT mixture for 20 minutes, while no false positives were observed (Table 3). Starting at an ambient temperature of 10°C, all triplicate reactions failed when incubated with either the 72.5% or 75% SAT heater (Table 3). However, when repeated with increased incubation times of 30 minutes, to compensate for reduced enzyme activity at suboptimal temperatures, all 3 reactions starting at ambient temperature of 10°C were positive with either concentration of SAT tested (Table 3).

**Table 3:** The performance of HIV RPA assays when incubated in SAT reactors at a range of ambient temperatures from 10°C to 30°C.

	72.5% SAT				75% SAT			
	HIV		NTC		HIV		NTC	
Temperature (°C)	20′	30′	20′	30′	20′	30′	20′	30′
10	0/3	3/3	0/1	0/1	0/3	3/3	0/1	0/1
15	3/3	3/3	0/1	0/1	3/3	3/3	0/1	0/1
20	3/3	3/3	0/1	0/1	3/3	3/3	0/1	0/1
25	3/3	3/3	0/1	0/1	3/3	3/3	0/1	0/1
30	3/3	3/3	0/1	0/1	3/3	3/3	0/1	0/1

SAT, Sodium acetate trihydrate; 20′–20 minutes total incubation time; 30′–30 minutes total incubation time; NTC - no template control; NTC, no template control.

### Analysis of daily and seasonal temperature data from Sub Saharan Africa

**Figure 3:**
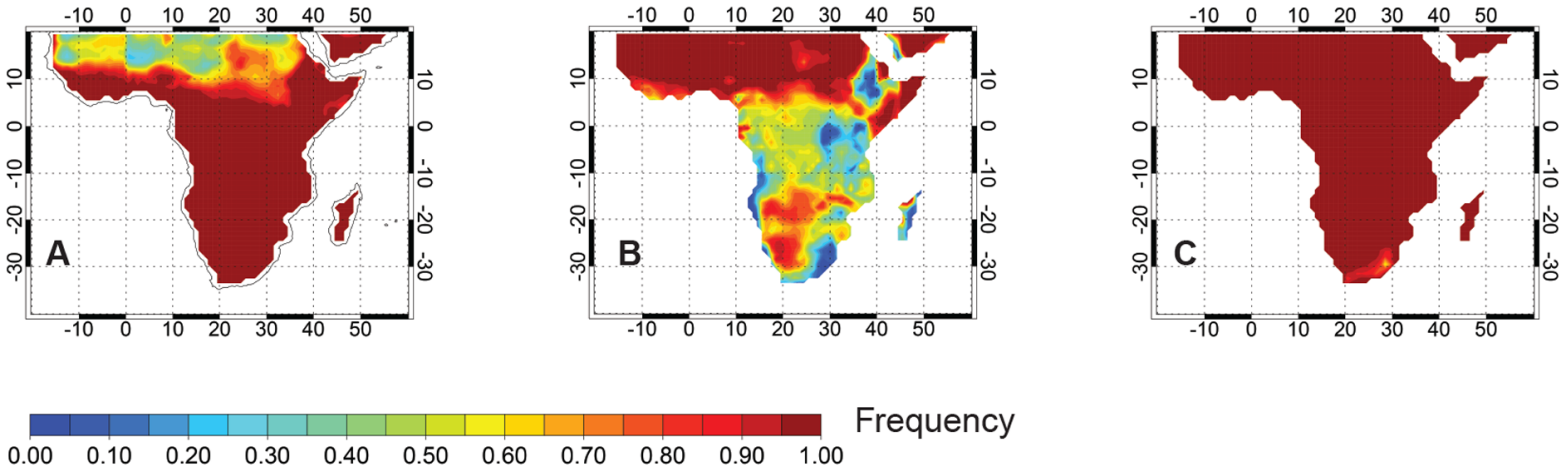
Maps depicting sub Saharan Africa and the frequency (fraction of days) on which mean afternoon (12–18 hr local time) surface air temperatures are below 43°C during the warm season (A), exceed 30°C in the warm season (B) and are above 10°C in the cold season (C).

To assess the viability of using RPA assays under ambient meteorological conditions, we analyzed an existing dataset of the surface air temperature in Africa between 2000–2010 [32]. Here we focus on temperatures recorded in the region of sub Saharan Africa (continental Africa south of 20°N) most afflicted by the HIV epidemic. Air temperature at any given site experiences significant variability on diurnal (day-night) to seasonal timescales. We distilled the data into 6 hourly intervals (0–6, 6–12, 12–18, 18–24 hour local time) and examine statistics for each of four seasons (Dec-Feb, Mar-May, June-Aug, Sept-Nov). For each 1×1° location in the dataset, the season with the warmest mean temperature is termed the *warm season* and the season with the coldest mean temperature is termed the *cold season*. The RPA assay tests were shown to work well for the temperature range 30°C–43°C. Air temperatures in sub Saharan Africa were found to rarely exceed the upper limit (43°C) even during the warm season and the afternoon (12–18 hr local time; Figure 3A). For example, in the harshest desert regions north of 10°N, mean afternoon temperatures are below 43°C on over 70% of days. South of 10°N, where the HIV incidence is greatest and population density is higher, afternoon warm season air temperatures are lower than 43°C on over 95% of days.

We examined the frequency at which the mean afternoon temperature exceeds 30°C during both the cold and warm seasons. During the cold season, the frequency of mean afternoon temperatures exceeding 30°C is lower than 10% in most locations. Exceptions include the desert regions north of 10°N where mean afternoon temperatures exceed 30°C on more than 50% of days during the cold season, and in the low-lying rainforest of the Congo Basin where mean afternoon temperatures exceed 30°C on 20–40% of days. During the warm season, a similar geographical pattern exists, but the frequency with which mean afternoon temperatures exceed 30°C is increased everywhere (Figure 3B). North of the equator during the warm season, mean afternoon temperatures exceed 30°C on over 50% of days. For most non-coastal locations south of 10°S, warm season mean afternoon temperatures exceed 30°C on approximately 60% of days. In the Congo Basin, warm season mean afternoon temperatures exceed 30°C on more than 40% of days. Exceptions include strongly elevated terrain (>2000 m) where mean afternoon temperatures rarely exceed 30°C. Analysis of the surface temperature data show that the mean afternoon air temperatures over most of sub-Saharan Africa exceed 10°C on nearly all days, even during the cold season (Figure 3C), so that a simple heating system should lead to successful RPA tests at most locations under almost all ambient environmental conditions.

## Discussion

RPA provides an attractive method for use in point of care diagnostics because it can be performed over a relatively broad range of incubation temperatures with no observed loss in performance when end point detection of the assay product was used. In this work we assessed the performance of a previously described HIV-1 RPA assay to reliably detect 10 copies of HIV DNA using ambient temperatures between 30°C–43°C, using the standard, commercially available formulation of RPA reagents. At ambient temperatures below 31°C, an exothermic chemical heater could be used to provide supplementary heat for optimal RPA performance to as low an ambient temperature of 10°C. These results demonstrate that, even in the absence of electricity or a battery dependent heater, RPA is significantly faster than other isothermal assays that are being developed as tools for NAAT based diagnostics in LRS.

The incubation time required was only 20 minutes at the higher temperatures, with an increase to 30 minute incubations necessary at the lower temperature of 30°C to compensate for reduced enzyme kinetics at suboptimal temperatures. Longer incubation times have been shown to produce positive results at incubation times below 30°C when used with other unrelated RPA assays but longer incubation times were not tested in this study as this would lead to a less acceptable time to result. To reinforce the plausibility of using ambient temperature for RPA incubation we performed an analysis of existing meteorological data for the sub Saharan Africa region, the center of the HIV epidemic, to determine the daily ambient temperature ranges in the warmest and coldest seasons. In the warm season, the entire region has surface temperatures that often exceed 30°C after noon, providing potential to incubate RPA assays without the need for a heater during this season in many areas within this region. In addition, pooled temperature data revealed that average temperatures were rarely observed to elevate above 43°C in this region, and therefore temperatures too high for optimal RPA are not commonly encountered. However, the analysis of seasonal meteorological data highlighted that many regions in sub Saharan Africa have ambient temperatures below 30°C, especially in elevated regions which represent a significant area of this landmass. From analysis of the meteorological data we also noted that ambient temperatures of less than 10°C are not commonly observed in this region. While this work focused on sub-Saharan Africa where the HIV epidemic is greatest and so is the primary intended market for the HIV diagnostic, there are also other LRS worldwide which would greatly benefit from better access to RPA based NAATs. While the climate might differ to those found in Sub Saharan Africa, many will have day time ambient temperatures that will allow for incubation using the approaches discussed herein.

To accommodate the lower temperatures observed we developed basic chemical heaters that can successfully incubate HIV-1 RPA assays even when the ambient temperature is as low as 10°C. These prototype SAT heaters were rechargeable by heating at 80°C for 20 minutes and cost less than $0.10 (USD) to make per prototype device. In areas where the temperatures fall below 10°C the SAT concentrations may be further adjusted to create a heater that will incubate the RPA reactions to within the required temperature ranges. Insulation of these heaters will be vital to minimize significant heat loss. For those with ambient temperatures above 45°C we would recommend that testing is held off until temperatures lie within in a more acceptable range. The SAT heater used in this study is only a prototype, and not the final design. It is anticipated that the final SAT heater device will be fully enclosed, with metal kinetic switch housed within the solution to initiate activation. The device can then be easily placed in a cup of boiled water to resolubilize the SAT before it is ready to be used again.

Ambient temperatures and SAT heaters are not the only instrument free options available. In addition, it is interesting to note that human body temperature is within the optimal temperature range for our HIV-1 RPA assay, and suggests that manual incubation may be possible. A simple device could be created, which could be placed under the arms or held to incubate the tube to approximately 35–37°C and allow for amplification to occur.

Overall, there is compelling evidence that RPA can be used for the application of high performance NAAT diagnosis of HIV-1 as well as other infectious diseases using either the ambient environmental temperature if above 30°C, or by using the most basic of heat sources for incubation when the ambient temperature is greater than 10°C. NAAT-based diagnostic tests in low resource settings face a variety of challenges, including access to adequate electrical power to run equipment or to recharge batteries, as many of these NAATs require prolonged incubation at elevated temperatures (>55°C) within a narrow range for optimum amplification. Typically this means that powered instrumentation is necessary to address precise incubation temperatures. However RPA does not have such stringent requirements. A previously described RPA assay to detect methicillin resistant Staphylococcus aureus was similarly noted to detect target DNA in a temperature range spanning from 25°C to 44°C, [16], therefore it is highly probable that this temperature range could be applied to any RPA based target. This relatively broad range of incubation temperatures suggests that RPA can be effectively performed using either ambient temperatures without a heat source or by using a simple electricity-free heat source supplied via an exothermic chemical reaction [29]. Here we demonstrate the potential benefit of using RPA in point-of-care diagnostics in settings where access to reliable electrical power is limited. As this technology does not require a precise temperature for incubation, ambient temperatures or a simple heating device can be used to achieve accurate and reproducible results, making it a powerful diagnostic tool.

## Supporting Information

Table S1 **Thermal profiles from preliminary screening of SAT concentrations of 50%–80% (w/v) after activation from an ambient temperature of 22°C.** Reactions were performed in insulated 50 mL tubes.
(DOCX)
