# Supplementary material for: Non-Instrumented Incubation of a Recombinase Polymerase Amplification Assay for the Rapid and Sensitive Detection of Proviral HIV-1 DNA
Source: PLoS One. 2014 Sep 29;9(9):e108189. doi: 10.1371/journal.pone.0108189 (PMC4180440; doi:10.1371/journal.pone.0108189)
Supplement: Table S1 — Thermal profiles from preliminary screening of SAT concentrations of 50%–80% (w/v) after activation from an ambient temperature of 22°C. Reactions were performed in insulated 50 mL tubes. (DOCX) [file pone.0108189.s001.docx]

**Table SI:** Thermal profiles from preliminary screen of SAT concentrations of 50%- 80% w/v carried out at ambient temperatures of 22 °C. Reactions were carried out in insulated 50ml tubes.

|  | 50 % SAT* | 60 % SAT | 70% SAT | 75% SAT | 80% SAT |
| --- | --- | --- | --- | --- | --- |
| Maximum Temp. (°C) | n/a | 26 | 35 | 41 | 46 |
| Temp. (°C) at 10 minutes | n/a | 26 | 34 | 39 | 45 |
| Temp. (°C) at 20 minutes | n/a | 26 | 34 | 38 | 43 |
| Temp. (°C) at 30 minutes | n/a | 26 | 33 | 37 | 42 |
| Temp. loss over 30 minutes (°C) | n/a | 0 | 2 | 4 | 4 |
| Time (sec) to reach max. temp. | n/a | 678 | 132 | 97 | 76 |
| Time (sec) to 95% of max. temp. | n/a | 54 | 15 | 8 | 20 |

SAT, Sodium acetate trihydrate.

*50% SAT solutions could not be activated at room temperature.
